# Characterization of Hip Fractures Among Adults With Schizophrenia in Ontario, Canada

**DOI:** 10.1001/jamanetworkopen.2023.10550

**Published:** 2023-04-28

**Authors:** Hina Ansari, Susan Jaglal, Angela M. Cheung, Paul Kurdyak

**Affiliations:** 1 Institute of Health Policy, Management, and Evaluation, Dalla Lana School of Public Health, University of Toronto, Toronto, Ontario, Canada; 2 Toronto Rehabilitation Institute, University Health Network, Toronto, Ontario, Canada; 3 Department of Physical Therapy, University of Toronto, Toronto, Ontario, Canada; 4 ICES, Toronto, Ontario, Canada; 5 Department of Medicine and Joint Department of Medical Imaging, University Health Network and Sinai Health System, University of Toronto, Toronto, Ontario, Canada; 6 Department of Psychiatry, Temerty Faculty of Medicine, University of Toronto, Toronto, Ontario, Canada; 7 Centre for Addiction and Mental Health, Toronto, Ontario, Canada

## Abstract

**Importance:**

Evidence suggests that individuals with schizophrenia are at an increased risk of hip fractures; however, the sex-specific burden of hip fractures among adults with schizophrenia has not been quantified and compared with the general population.

**Objective:**

To describe sociodemographic and clinical characteristics of patients with hip fracture and schizophrenia and to quantify their sex-specific annual hip fracture rates relative to those without schizophrenia.

**Design, Setting, and Participants:**

This repeated population-based, cross-sectional study leveraged multiple individually linked health administrative databases for patients in Ontario, Canada. We included patients aged 40 to 105 years with hip fracture–related hospitalization between April 1, 2009, and March 31, 2019. Statistical analysis was performed between November 2021 and February 2023.

**Exposure:**

Schizophrenia diagnosis, ascertained using a validated algorithm.

**Main Outcomes and Measures:**

The main outcome was sex-specific age-standardized annual hip fracture rate per 10 000 individuals and annual percent change in age-standardized rates. Rates were direct adjusted to the 2011 Ontario population, and joinpoint regression analysis was performed to evaluate annual percent change.

**Results:**

We identified 117 431 hip fracture records; of these, there were 109 908 index events. Among the 109 908 patients with hip fracture, 4251 had schizophrenia and 105 657 did not. Their median age was 83 years (IQR, 75-89 years), and 34 500 (31.4%) were men. Patients with hip fracture and schizophrenia were younger at the index event compared with those without schizophrenia. Men had a median age of 73 vs 81 years (IQR, 62-83 vs 71-87 years; standardized difference, 0.46), and women had a median age of 80 vs 84 years (IQR, 71-87 vs 77-89 years; standardized difference, 0.32). A higher proportion of patients with vs without schizophrenia had frailty (53.7% vs 34.2%; standardized difference, 0.40) and previous fragility fractures (23.5% vs 19.1%; standardized difference, 0.11). The overall age-standardized rate per 10 000 individuals with vs without schizophrenia was 37.5 (95% CI, 36.4 to 38.6) vs 16.0 (95% CI, 15.9 to 16.1). Age-standardized rates were 3-fold higher in men with vs without schizophrenia (31.0 [95% CI, 29.5 to 32.6] vs 10.1 [95% CI, 10.0 to 10.2]) and more than 2-fold higher in women with vs without schizophrenia (43.4 [95% CI, 41.9 to 44.9] vs 21.4 [95% CI, 21.3 to 21.6]). Overall, joinpoint regression analysis identified a steady annual decrease of 0.7% (95% CI, −1.1% to −0.3%) in age-standardized rates for both study groups.

**Conclusions and Relevance:**

The findings of this cross-sectional study suggest that individuals with schizophrenia experience an earlier age of onset and considerably higher rate of hip fractures compared with the general population, with implications for targeted fracture prevention and optimization of clinical bone health management over the course of their psychiatric illness.

## Key Points

**Question:**

How do hip fracture rates among individuals with schizophrenia compare with the general population?

**Findings:**

In this cross-sectional study of 109 908 patients with hip fracture in Ontario, Canada, the annual age-standardized rate was 38 vs 16 hip fractures per 10 000 individuals with vs without schizophrenia, with greater relative differences observed among men. Patients with schizophrenia and hip fracture were younger, were more likely to be frail, and were more likely to have previous fragility fractures.

**Meaning:**

These findings suggest that individuals with schizophrenia experience an earlier onset and substantially higher burden of hip fractures, with implications for targeted fracture prevention and optimization of bone health management over the course of their psychiatric illness.

## Teaser

This cross-sectional study examines the sociodemographic and clinical characteristics of patients with hip fractures who have schizophrenia and compares them with those of patients with hip fractures who do not have schizophrenia.

## Introduction

Hip fractures are associated with loss of functional independence, disability, and institutionalization, resulting in devastating consequences for patients and their health care systems.^1,2,3,4^ Patients with hip fracture also face a substantial excess risk of mortality,^5^ which can persist for many years after the sentinel event,^6,7^ with men being more likely to experience adverse outcomes than women.^8,9,10^ Although incidence rates may be declining in some jurisdictions, a simultaneous increase in the absolute number of hip fracture cases is a concern.^11,12^ Prevention of hip fractures is a well-recognized public health priority worldwide; identifying vulnerable subpopulations who may experience a higher burden of illness is critical to informing targeted preventative strategies.

Individuals living with schizophrenia experience a higher prevalence of hip fracture–related risk factors, which includes antipsychotic medications,^13,14,15,16,17,18^ lifestyle-related factors (smoking,^19,20^ reduced physical activity,^21,22,23^ heavy alcohol intake,^24,25^ vitamin D deficiency^26,27^), low bone mineral density,^28^ and diabetes.^29,30^ Although there is a growing evidence base documenting the association between schizophrenia and fracture risk,^31,32,33,34,35,36,37^ little is known about the burden of hip fractures specifically.^32,33,34,36^ The age- and sex-specific incidence of hip fractures among individuals with schizophrenia has not been determined, particularly within the North American context. Jurisdiction-specific findings are warranted, given the wide variation observed in the burden of hip fractures globally.^38^ Furthermore, existing population-based analyses that have only leveraged psychiatric hospital records for ascertainment of schizophrenia may have missed cases managed entirely in ambulatory settings. This may bias study samples toward more severe schizophrenia cases and may possibly overestimate the burden of hip fractures associated with schizophrenia. Given that people with schizophrenia experience substantially lower life expectancy relative to the general population,^39,40^ it is a priority to understand and address preventable health disparities in this disadvantaged subpopulation.^41^

The purpose of this study was to quantify and compare trends and characteristics of hip fractures sustained between fiscal years 2009 and 2018 in Ontario, Canada, among all adults aged 40 years or older living with schizophrenia vs the general population. We had 2 specific study objectives: (1) to compare patients with hip fracture and schizophrenia relative to those without schizophrenia on sociodemographic and clinical characteristics and (2) to report on annual sex-specific rates of hip fractures between individuals with and without schizophrenia.

## Methods

The data used in this cross-sectional study are housed at ICES, a prescribed entity authorized to collect and use health care and demographic data for health system evaluation and improvement. Projects that use data collected by ICES under section 45 of Ontario’s Personal Health Information Protection Act (PHIPA), and use no other data, are exempt from research ethics board review. The use of the data in this project is authorized under section 45 of the PHIPA and approved by the ICES Privacy and Legal Office. This study followed the Reporting of Studies Conducted Using Observational Routinely Collected Health Data (RECORD) reporting guideline.

### Study Setting and Participants

In this cross-sectional study, we examined adults aged 40 to 105 years who sustained a hip fracture–related hospitalization between April 1, 2009, and March 31, 2019, in Ontario, Canada. The province of Ontario has 14 million residents and a universal single-payer health care system that covers medically necessary hospital and physician services. We identified all hip fracture hospitalizations using the following *International Statistical Classification of Diseases, Tenth Revision, Canada* (*ICD-10-CA*) discharge diagnosis codes: S720.0 (head/neck of femur), S720.1 (pertrochanteric fracture), and S720.2 (subtrochanteric fracture). To ensure that all hip fracture–related hospitalizations were captured, we included hip fractures that were coded as the most responsible diagnosis or a secondary diagnosis. Patient records with an invalid identifier, age younger than 40 or older than 105 years at the time of admission, and those belonging to non-Ontario residents were excluded. We chose 40 years as our minimum age cutoff, given that individuals with fragility fractures after this age are recognized as a distinct clinical group in terms of bone health management, and this threshold has also been used for widescale fracture surveillance in our jurisdiction.^10^

### Data Sources

This study leveraged multiple health administrative ICES data sets, which were linked using unique encoded identifiers and analyzed at ICES. These data sets were as follows: the Canadian Institute of Health Information Discharge Abstract Database (for data relevant to inpatient hospitalizations at general acute care hospitals), the National Ambulatory Care Reporting System (for emergency department visit data), the Ontario Health Insurance Plan (OHIP) database (for physician service claims), the Ontario Mental Health Reporting System (OMHRS) database (for hospitalizations to adult psychiatric beds), the Registered Persons Data Base (for patient demographic data and vital statistics), the Ontario Marginalization Index (for area-level marginalization indices), the Continuing Care Reporting System Long-term Care and the Ontario Drug Benefits databases (to determine transfer from a long-term care [LTC] facility), the Client Agency Program Enrolment database (to determine access to primary care), and Statistics Canada Census 2011 and 2016 estimates (to derive neighborhood characteristics). Age- and sex-stratified population denominators for each study year were also obtained from Statistics Canada Census estimates.

### Exposure Ascertainment

We used a validated algorithm to identify cases with preexisting schizophrenia based on *ICD-10-CA* (F20, F25, and F29), OMHRS (295), and OHIP (295 and 298) diagnosis codes. Ascertainment was on the basis of a single psychiatric hospital admission within 12 months or 3 physician visits within 36 months prior to fracture (96.5% sensitivity and 57.1% specificity).^42^

### Measurement of Sociodemographic and Clinical Characteristics

Neighborhood income quintiles and the Ontario Marginalization Index were used to measure socioeconomic disadvantage and marginalization.^43^ Area-based income quintile was detected at the level of dissemination areas, based on the smallest census-defined regions. Rurality was measured using the Rurality Index of Ontario, whereby scores less than 39 refer to urban areas and scores of 40 or greater refer to rural areas.^44^ We ascertained LTC residents using a combination of 1 drug claim and 2 physician billing codes associated with LTC-dwelling status made within 180 days prior to the hip fracture episode.^45^ The patient’s history of previous fractures was ascertained using a look-back window of up to 10 years, counting only study-defined fragility fractures sustained after age 40 years (eTable 1 in Supplement 1). A previous fragility fracture after age 40 years is the strongest risk factor for subsequent fractures.^46^ The John Hopkins ACG System Aggregated Diagnosis Groups (ADGs) were used to assess comorbidities, as they have been validated specifically among schizophrenia populations.^47^ Medical ADGs were reported separately from psychosocial (mental health-related) ADGs. The John Hopkins ACG System’s multidimensional frailty indicator was used to characterize frailty, an established risk factor for fractures.^48,49^ This instrument was designed for health administrative data and has been successfully used in Ontario to evaluate outcomes at the population level.^50,51,52,53^ It captures patients with a diagnosis falling within any of the following frailty-associated clusters (representing 81 diagnosis codes): malnutrition, dementia, impaired vision, ulcer, incontinence, weight loss, falls, difficulty walking, poverty, and barriers in access to care.^54,55^ Due to the ACG system’s proprietary nature, we were unable to provide the specific diagnosis codes. Our look-back window to assess all comorbidities was 2 years prior to the hip fracture event. Patients who had surgery for their hip fracture were identified using a combination of physician billing and intervention codes (eTable 1 in Supplement 1).^56^

### Statistical Analysis

Differences in sociodemographic and clinical characteristics were compared using standardized differences, with values greater than 0.1 indicating meaningful significance. Differences were assessed using a cohort restricted to the first hip fracture episode per individual during the study period (referred to as the index episode from here onward). The percentage of missing data for each variable was also reported.

Annual unadjusted crude fracture rates per 10 000 person-years with 95% CIs were calculated for the overall population, by sex and study-defined age groups. Rates were direct adjusted to the 2011 age structure of the Ontario population to allow for comparisons across time, and between those with and without schizophrenia. Confidence intervals for age-standardized rates were calculated using the gamma method.^57^ Denominators for the Ontario population were obtained from Statistics Canada population census files. Joinpoint regression analysis was performed to evaluate the annual percent change (APC) in annual hip fracture rates from fiscal years 2009 to 2018. Annual percentage changes, which are useful for comparing populations expected to have different rates, were computed using generalized linear models assuming a Poisson distribution. Tests for parallelism were performed to evaluate whether the rate of change differed across time between those with vs without schizophrenia. The software also checked for the presence of time points where a statistically significant change in trend (joinpoint) may have occurred, starting with a model with the minimum number of joinpoints, and successively testing whether 1 or more joinpoints should be added.^58^

In sensitivity analyses, we recalculated crude rates by removing any records for hip fracture that occurred within 6 months of the index hip fracture event, as these events may be related.^59^ The purpose of this sensitivity analysis was to check if removing subsequent hospitalizations would change the relative differences in rates between those with vs without schizophrenia.

SAS Enterprise Guide, version 9.4 (SAS Institute Inc), was used to conduct all analyses, except for trend analyses of APCs, which were conducted using Joinpoint Regression software, version 4.9.1.0 (National Cancer Institute). Statistical analysis was performed between November 2021 and February 2023.

## Results

In this 10-year cross-sectional study of adults aged 40 to 105 years residing in Ontario, 117 431 hip fracture–related hospitalizations were identified. Of these, 109 908 were index hip fracture hospitalizations, with a median patient age of 83 years (IQR, 75-89 years) (Table 1). A total of 34 500 patients (31.4%) were men and 75 408 (68.6%) were women. There were 4251 (3.9%) index hip fractures among individuals with schizophrenia.

**Table 1.** Sociodemographic and Clinical Characteristics of Individuals Aged 40 Years or Older With vs Without Schizophrenia Who Sustained an Index Hip Fracture During Fiscal Years 2009 to 2018, in Ontario, Canada^a^

Characteristic	Full cohort (N = 109 908)	Patients without schizophrenia (n = 105 657)	Patients with schizophrenia (n = 4251)	Standardized difference
**Sociodemographic**				
Sex				
Women	75 408 (68.6)	72 645 (68.8)	2763 (65.0)	0.08
Men	34 500 (31.4)	33 012 (31.2)	1488 (35.0)	0.08
Age, y				
Mean (SD)	80.53 (11.3)	80.70 (11.2)	76.35 (12.4)	0.37
Median (IQR)	83 (75-89)	83 (75-89)	78 (68-86)	0.37
Rural or small town				
No	94 609 (86.1)	90 789 (85.9)	3820 (89.9)	0.12
Yes	14 832 (13.5)	14 420 (13.6)	412 (9.7)	0.12
Missing	467 (0.4)	448 (0.4)	19 (0.4)	0.00
LTC-dwelling resident (prefracture)	17 367 (15.8)	15 923 (15.1)	1444 (34.0)	0.45
Neighborhood income quintile				
1 (Lowest)	26 384 (24.0)	25 031 (23.7)	1353 (31.8)	0.18
2	23 218 (21.1)	22 339 (21.1)	879 (20.7)	0.01
3	20 796 (18.9)	20 079 (19.0)	717 (16.9)	0.06
4	19 815 (18.0)	19 154 (18.1)	661 (15.5)	0.07
5 (Highest)	18 931 (17.2)	18 322 (17.3)	609 (14.3)	0.08
Missing	764 (0.7)	732 (0.7)	32 (0.8)	0.01
Instability quintile				
1 (Lowest)	11 127 (10.1)	10 814 (10.2)	313 (7.4)	0.10
2	15 688 (14.3)	15 219 (14.4)	469 (11.0)	0.10
3	19 425 (17.7)	18 828 (17.8)	597 (14.0)	0.10
4	24 721 (22.5)	23 811 (22.5)	910 (21.4)	0.03
5 (Highest)	37 549 (34.2)	35 669 (33.8)	1880 (44.2)	0.22
Missing	1398 (1.3)	1316 (1.2)	82 (1.9)	0.06
Dependency quintile				
1 (Lowest)	11 236 (10.2)	10 745 (10.2)	491 (11.6)	0.04
2	14 507 (13.2)	13 885 (13.1)	622 (14.6)	0.04
3	17 105 (15.6)	16 459 (15.6)	646 (15.2)	0.01
4	21 095 (19.2)	20 323 (19.2)	772 (18.2)	0.03
5 (Highest)	44 567 (40.5)	42 929 (40.6)	1638 (38.5)	0.04
Missing	1398 (1.3)	1316 (1.2)	82 (1.9)	0.06
Deprivation quintile				
1 (Lowest)	18 528 (16.9)	17 951 (17.0)	577 (13.6)	0.10
2	20 567 (18.7)	19 901 (18.8)	666 (15.7)	0.08
3	21 079 (19.2)	20 303 (19.2)	776 (18.3)	0.03
4	22 611 (20.6)	21 751 (20.6)	860 (20.2)	0.01
5 (Highest)	25 725 (23.4)	24 435 (23.1)	1290 (30.3)	0.16
Missing	1398 (1.3)	1316 (1.2)	82 (1.9)	0.06
Ethnic concentration quintile				
1 (Lowest)	24 147 (22.0)	23 434 (22.2)	713 (16.8)	0.14
2	24 346 (22.2)	23 521 (22.3)	825 (19.4)	0.07
3	21 495 (19.6)	20 649 (19.5)	846 (19.9)	0.01
4	20 323 (18.5)	19 409 (18.4)	914 (21.5)	0.08
5 (Highest)	18 199 (16.6)	17 328 (16.4)	871 (20.5)	0.11
Missing	1398 (1.3)	1316 (1.2)	82 (1.9)	0.06
**Clinical or health care**				
Received hip fracture surgery	102 454 (93.2)	98 553 (93.3)	3901 (91.8)	0.06
Index hip fracture episode was coded as MRDx	104 730 (95.3)	100 747 (95.4)	3983 (93.7)	0.07
Type of index hip fracture				
Head/neck of femur	53 150 (48.4)	51 151 (48.4)	1999 (47.0)	0.03
Pertrochanteric	46 968 (42.7)	45 144 (42.7)	1824 (42.9)	0.00
Subtrochanteric	5621 (5.1)	5432 (5.1)	189 (4.4)	0.03
Other concurrent fracture with index hip fracture				
Vertebral fracture	532 (0.5)	505 (0.5)	27 (0.6)	0.02
Pelvis fracture	1016 (0.9)	966 (0.9)	50 (1.2)	0.03
Upper limb fracture	3903 (3.6)	3761 (3.6)	142 (3.3)	0.01
History of hip fracture (5-y look-back and patient aged ≥40 y)	1415 (1.3)	1350 (1.3)	65 (1.5)	0.02
History of nonhip fragility fracture (10-y look-back and patient aged ≥40 y at the time)	21 221 (19.3)	20 223 (19.1)	998 (23.5)	0.11
History of either a hip or nonhip fragility fracture	22 148 (20.2)	21 107 (20.0)	1041 (24.5)	0.11
Frailty (from John Hopkins System)	38 429 (35.0)	36 148 (34.2)	2281 (53.7)	0.40
No. of medical ADGs				
Mean (SD)	8.37 (3.73)	8.36 (3.72)	8.55 (4.07)	0.05
Median (IQR)	8 (6-11)	8 (6-11)	8 (5-12)	0.04
No. of psychosocial ADGs				
0	51 436 (46.8)	50 848 (48.1)	588 (13.8)	0.80
1	38 746 (35.3)	37 163 (35.2)	1583 (37.2)	0.04
2	17 306 (15.7)	15 623 (14.8)	1683 (39.6)	0.58
3	2420 (2.2)	2023 (1.9)	397 (9.3)	0.33
Rostered to a primary care provider^b^				
0	4780 (4.3)	4437 (4.2)	343 (8.1)	0.16
1	87 802 (79.9)	84 787 (80.2)	3015 (70.9)	0.22
2	17 326 (15.8)	16 433 (15.6)	893 (21.0)	0.14

Abbreviations: ADG, aggregated diagnosis group; LTC, long-term care; MRDx, most responsible diagnosis.

^a^ Unless indicated otherwise, values are presented as No. (%) of patients.

^b^ Categorized as follows: 0, not rostered; 1, rostered; 2, virtually rostered.

### Sociodemographic Characteristics of Patients With Schizophrenia and Hip Fracture

Patients with hip fracture with vs without schizophrenia were younger at the time of index hip fracture. Men had a median age of 73 vs 81 years (IQR, 62-83 vs 71-87 years; standardized difference, 0.46), and women had a median age of 80 vs 84 years (IQR, 71-87 vs 77-89 years; standardized difference, 0.32) (eTable 2 in Supplement 1). Women comprised approximately 65.0% vs 68.8% of patients with vs without schizophrenia (standardized difference, 0.08). A higher proportion of patients with hip fracture with vs without schizophrenia came from LTC settings (34.0% vs 15.1%; standardized difference, 0.45). Patients with hip fracture with vs without schizophrenia were more likely to be within the lowest neighborhood-level income quintile (31.8% vs 23.7%; standardized difference, 0.18), highest instability quintile (44.2% vs 33.8%; standardized difference 0.22), highest deprivation quintile (30.3% vs 23.1%; standardized difference, 0.16), and highest ethnic concentration quintile (20.5% vs 16.4%; standardized difference, 0.11).

### Clinical Characteristics of Patients With Schizophrenia and Hip Fracture

Individuals with schizophrenia were more likely to have had a previous fragility fracture compared with those without schizophrenia (23.5% vs 19.1%; standardized difference, 0.11), with women reporting higher rates of previous fractures than men (eTable 2 in Supplement 1). There was less than a 10% standardized difference in the proportion of those with vs without schizophrenia for type of hip fracture sustained, whether they received hip fracture surgery, and whether they had sustained another simultaneous fracture at a different site. A higher proportion of patients with hip fracture with vs without schizophrenia were identified as having frailty (53.7% vs 34.2%; standardized difference, 0.40). We observed less than a 10% standardized difference in the mean number of medical diagnoses. Finally, patients with hip fracture with vs without schizophrenia were less likely to be rostered to a primary care provider (91.9% vs 95.8%; standardized difference, 0.16).

### Crude Rates

Table 2 provides hip fracture numbers and crude rates, by sex, for each year of observation, for those with and without schizophrenia. The crude annual rate per 10 000 (95% CI) among individuals with schizophrenia was 41.4 (40.2 to 42.6) compared with 16.5 (16.4 to 16.6) among those without schizophrenia. The crude annual rate (95% CI) among men with schizophrenia was 30.1 (28.6 to 31.6) compared with 51.8 (49.9 to 53.6) among women with schizophrenia.

**Table 2.** Annual Numbers and Crude Rates of Hip Fracture per 10 000 Individuals Aged 40 Years or Older With vs Without Schizophrenia During Fiscal Years 2009 to 2018, in Ontario, Canada

Year	Individuals with schizophrenia			Individuals without schizophrenia		
	No. of hip fracture events observed	Population (≥40 y)	Crude rate/10 000 (95% CI)	No. of hip fracture events observed	Population (≥40 y)	Crude rate/10 000 (95% CI)
Men						
2009	153	47 423	32.3 (27.4-37.8)	2969	3 055 878	9.7 (9.4-10.1)
2010	158	48 284	32.7 (27.8-38.2)	3060	3 111 395	9.8 (9.5-10.2)
2011	148	51 156	28.9 (24.5-34.0)	3107	3 163 836	9.8 (9.5-10.2)
2012	130	49 867	26.1 (21.8-31.0)	3347	3 213 402	10.4 (10.1-10.8)
2013	169	50 607	33.4 (28.6-38.8)	3594	3 261 101	11.0 (10.7-11.4)
2014	139	56 275	24.7 (20.8-29.2)	3574	3 302 406	10.8 (10.5-11.2)
2015	160	56 938	28.1 (23.9-32.8)	3523	3 341 285	10.5 (10.2-10.9)
2016	177	60 438	29.3 (25.1-33.9)	3667	3 384 094	10.8 (10.5-11.2)
2017	180	58 436	30.8 (26.5-35.7)	3933	3 429 174	11.5 (11.1-11.8)
2018	205	59 183	34.6 (30.1-39.7)	4066	3 473 065	11.7 (11.4-12.1)
All	1619	538 607	30.1 (28.6-31.6)	34 840	32 735 636	10.6 (10.5-10.8)
Women						
2009	290	53 355	54.4 (48.3-61.0)	7135	3 314 621	21.5 (21.0-22.0)
2010	300	54 342	55.2 (29.1-61.8)	7352	3 375 921	21.8 (21.3-22.3)
2011	269	56 896	47.3 (41.8-53.3)	7455	3 433 529	21.7 (21.2-22.2)
2012	307	56 132	54.7 (48.7-61.2)	7652	3 487 153	21.9 (21.5-22.4)
2013	313	56 998	54.9 (49.0-61.4)	8159	3 540 976	23.0 (22.5-23.6)
2014	317	60 289	52.6 (47.0-58.7)	7898	3 590 257	22.0 (21.5-22.5)
2015	338	61 050	55.4 (49.6-61.6)	7809	3 635 564	21.5 (21.0-22.0)
2016	310	63 928	48.5 (43.2-54.2)	8029	3 685 029	21.8 (21.3-22.3)
2017	295	62 777	47.0 (41.8-52.7)	8133	3 738 387	21.8 (21.3-22.2)
2018	312	63 630	49.0 (43.7-54.8)	8299	3 789 129	21.9 (21.4-22.4)
All	3051	589 397	51.8 (49.9-53.6)	77 921	35 590 566	21.9 (21.7-22.1)
Men and women						
2009	443	100 778	44.0 (40.0-48.3)	10 104	6 370 499	15.9 (15.6-16.2)
2010	458	102 626	44.6 (40.6-48.9)	10 412	6 487 316	16.0 (15.7-16.4)
2011	417	108 052	38.6 (35.0-42.5)	10 562	6 597 365	16.0 (15.7-16.3)
2012	437	105 999	41.2 (37.5-45.3)	10 999	6 700 555	16.4 (16.1-16.7)
2013	482	107 605	44.8 (40.9-49.0)	11 753	6 802 077	17.3 (17.0-17.6)
2014	456	116 564	39.1 (35.6-42.9)	11 472	6 892 663	16.6 (16.3-17.0)
2015	498	117 988	42.2 (38.6-46.1)	11 332	6 976 849	16.2 (15.9-16.5)
2016	487	124 366	39.2 (35.8-42.8)	11 696	7 069 123	16.5 (16.3-16.9)
2017	475	121 213	39.2 (35.7-42.9)	12 066	7 167 561	16.8 (16.5-17.1)
2018	517	122 813	42.1 (38.5-45.9)	12 365	7 262 194	17.0 (16.7-17.3)
All	4670	1 128 004	41.4 (40.2-42.6)	112 761	68 326 202	16.5 (16.4-16.6)

Table 3 presents the mean sex-specific crude annual hip fracture rate by age group. Within every age group (for both men and women), crude rates were higher among those with vs without schizophrenia. The relative difference in crude rates was greatest in the youngest age groups and decreased with increasing age. In sensitivity analyses (eTable 3 in Supplement 1), we observed that removal of hip fracture hospitalizations within 6 months of the index event did not meaningfully change the relative differences in crude rates from those presented in Table 3 of our main analyses.

**Table 3.** Distribution of Hip Fracture Events and Crude Hip Fracture Rate of Individuals Aged 40 Years and Over, by Age Group and Sex, for Those With vs Without Schizophrenia, Ontario, Canada

Age, y	Individuals with schizophrenia			Individuals without schizophrenia		
	No. of hip fracture events observed	Population (≥40 y)	Crude rate/10 000 (95% CI)	No. of hip fracture events observed	Population (≥40 y)	Crude rate/10 000 (95% CI)
Men						
40-54	163	266 551	6.1 (5.2-7.1)	2009	14 858 749	1.4 (1.3-1.4)
55-64	305	143 853	21.2 (18.9-23.7)	3445	8 564 360	4.0 (3.9-4.2)
65-79	592	93 738	63.2 (58.2-68.5)	10 283	7 175 673	14.3 (14.1-14.6)
≥80	559	34 467	162.2 (149-176.2)	19 103	2 136 854	89.4 (88.1-90.7)
All	1619	538 609	30.1 (28.6-31.6)	38 840	32 735 636	10.6 (10.5-10.8)
Women						
40-54	83	223 688	3.7 (3.0-4.6)	1360	15 101 811	0.9 (0.9-1.0)
55-64	301	155 886	19.3 (17.2-21.6)	4305	8 943 473	4.8 (4.7-5.0)
65-79	1063	133 291	79.8 (75.0-84.7)	19 123	8 108 380	23.6 (23.3-23.9)
≥80	1604	76 530	209.6 (199.5-220.1)	53 133	3 436 902	154.6 (153.3-155.9)
All	3051	589 395	51.8 (49.9-53.6)	77 291	35 590 566	21.9 (21.7-22.1)
Men and women						
40-54	246	490 239	5.0 (4.4-5.7)	3369	29 960 560	1.1 (1.1-1.2)
55-64	606	299 739	20.2 (18.6-21.9)	7750	17 507 833	4.4 (4.3-4.5)
65-79	1655	227 029	72.9 (69.4-76.5)	29 406	15 284 053	19.2 (19.0-19.5)
≥80	2163	110 997	194.9 (186.7-203.3)	72 236	5 573 756	129.6 (128.7-130.6)
All	4670	1 128 004	41.4 (40.2-42.6)	112 761	68 326 202	16.5 (16.4-16.6)

### Age-Standardized Rates and Annual Percent Change

The mean age-standardized rate per 10 000 individuals (95% CI) with vs without schizophrenia over the 10-year period was 31.0 (29.5 to 32.6) vs 10.1 (10.0 to 10.2) for men, 43.4 (41.9 to 44.9) vs 21.4 (21.3-21.6) for women, and 37.5 (36.4 to 38.6) vs 16.0 (15.9 to 16.1) overall (Figure). The error bars indicate 95% CIs, which did not overlap in the sex-specific comparisons presented.

**Figure.**
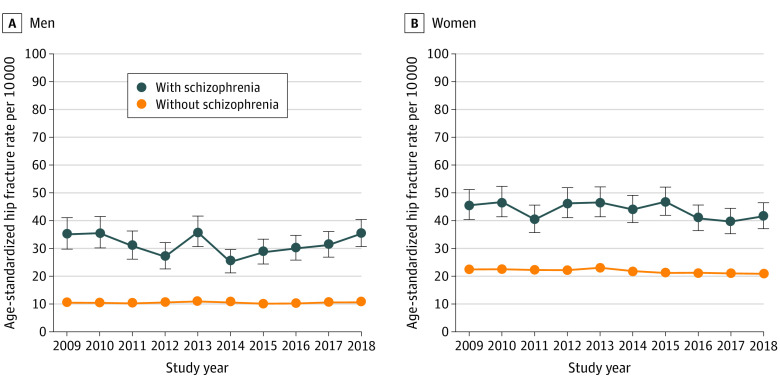
Annual Age-Standardized Hip Fracture Rates per 10 000 Individuals With vs Without Schizophrenia by Sex Rates were direct adjusted to the 2011 age structure of adults aged 40 years or older in Ontario, Canada. Error bars indicate 95% CIs. Study years represent fiscal years (eg, April 1, 2009, to March 31, 2019).

Joinpoint regression analysis of the APC demonstrated an annual decrease of 0.7% (95% CI, −1.1% to −0.3%) in age-standardized hip fracture rates among those with and without schizophrenia (Table 4). Tests for parallelism failed to reject the null hypothesis that the 2 rates of change were equal, comparing those with and without schizophrenia. The model without any joinpoints was chosen as the final model, demonstrating a steady change over the study period.

**Table 4.** Annual Percent Change in Age-Standardized Hip Fracture Rates by Sex Using Joinpoint Regression Analysis

Sex	Fiscal years	APC (95% CI)	Test for parallelism
All	2009-2018	−0.7 (−1.1 to −0.3)^a^	FTR
Women	2009-2018	−1.0 (−1.3 to −0.6)^a^	FTR
Men	2009-2018	0.0 (−0.6 to 0.6)	FTR

Abbreviations: APC, annual percent change; FTR, failed to reject.

^a^ Significantly different from 0 at the α = .05 level.

## Discussion

This cross-sectional study highlights several key similarities and differences in patient characteristics and trends among patients with hip fracture with and without schizophrenia using population-level data spanning 10 years. Patients with hip fracture and schizophrenia were notably younger at the time of their index fracture, and they were more likely to present with preexisting frailty and previous fragility fractures. Although there was a 0.7% annual decrease over the study period, age-standardized hip fracture rates for individuals with schizophrenia were consistently and substantially higher than those of the general population across all years of observation.

The absolute age-standardized hip fracture rates were higher for women than men with schizophrenia, similar to the trend observed in the general population. On average, age-standardized rates were 3 times higher in men with vs without schizophrenia and more than 2 times higher in women with vs without schizophrenia. Age-standardized rates for men with schizophrenia surpassed the observed rates for women in the general population. Furthermore, we highlight that the early onset of hip fractures was more pronounced among men with schizophrenia, whereby approximately one-third of male patients with hip fracture and schizophrenia were aged younger than 65 years at the time of index event. Our findings about male patients with hip fracture and schizophrenia are of particular concern. First, sex-based disparities in bone health management are well documented in the broader population, whereby men are less likely to receive evidence-based care.^10,60,61^ Moreover, men are also more likely to have worse outcomes after hip fractures, with an increased risk of mortality.

The mechanisms underlying the early onset of hip fractures among individuals with schizophrenia need to be further investigated, with careful attention to sex-specific differences in etiology. The substantial magnitude of the hip fracture burden that we observed in our study group with schizophrenia is likely a result of a multitude of complex risk factors acting over the course of the lifetime. Schizophrenia is typically diagnosed between the ages of 18 and 35 years, with several studies suggesting that men are more likely to be diagnosed earlier than women.^62^ The age of onset for schizophrenia may overlap with a critical stage of bone mass development.^63^ Furthermore, it has been suggested that initiation of antipsychotic medications at this stage may potentially interfere with optimal bone mass development, predisposing these individuals to fracture risk early in life, and this predisposition can be further intensified by the early accumulation of other clinical and lifestyle risk factors.^15,28^

Strengths of this study include the use of large population-level data spanning a 10-year period, with a diverse general population-based comparison group. To our knowledge, this study is the first to report sex-specific age-standardized estimates of annual hip fracture rates in a North American population with schizophrenia, leveraging a validated algorithm for case ascertainment of schizophrenia^42^ and methods that are aligned with standardized hip fracture surveillance efforts.^10,59,64^ Rate estimates for our general population comparison group demonstrated consistency with a recent evaluation of hip fracture trends among all Canadians aged 40 years or older during the period 2000 to 2016.^10^ Our approach can be replicated within other countries with similar data quality.

### Limitations

Our findings should be interpreted within the context of certain limitations. First, we acknowledge that our assessment of frailty was operationalized using a multidimensional instrument, and that the prevalence of frailty among patients with hip fracture and schizophrenia may vary with other methods of measurement. Furthermore, although our findings demonstrate a disparity in hip fracture rates among men and women with schizophrenia compared with the general population, the analysis was not designed to prove causality or to establish schizophrenia as an independent clinical risk factor for the occurrence of hip fractures. Further research is needed to elucidate causal mechanisms underlying our findings and to inform tailored preventative strategies.^65^ This would require more granular-level data on characteristics such as weight, smoking history, bone density, parental hip fractures, vision (eg, depth perception, contrast sensitivity), caffeine intake, and physical activity, all of which can confer a higher risk for hip fractures^9^ but also tend to be unavailable in routinely collected health administrative data sources. We did not investigate the contribution of antipsychotic medications or the degree to which different antipsychotic medications may explain variability in hip fracture burden. This is an important area of future study. Furthermore, our exposure group captured only those with schizophrenia specifically; however, it is important to understand how hip fracture rates may vary in individuals with other types of mental illness. Nonetheless, our findings have important implications for evaluating osteoporosis management and post–hip fracture outcomes for a disadvantaged subpopulation.

## Conclusions

The findings of this cross-sectional study suggest that individuals with schizophrenia may experience earlier onset and a substantially higher annual age-standardized rate of hip fractures compared with the general population, with greater relative differences among men. Patients with hip fracture and schizophrenia are also more likely to have previous fragility fractures and meet criteria for frailty. These findings have important implications for targeted fracture prevention and optimizing clinical management of bone health in a vulnerable population. Further research is needed to elucidate sex-specific causal mechanisms underlying the increased burden and to evaluate outcomes and care for patients with schizophrenia who sustain a hip fracture.

## Notes

Supplement 1. **eTable 1.** Diagnosis and Physician Billing Codes
eReferences
**eTable 2.** Sex-Stratified Comparison of Sociodemographic and Clinical Characteristics of Individuals Aged 40 Years or Older, With Versus Without Schizophrenia, Who Sustained an Index Hip Fracture During Fiscal Years 2009 to 2018 in Ontario (N = 109 908) **eTable 3.** Sensitivity Analyses: Distribution of Hip Fracture Events and Crude Hip Fracture Rate of Individuals Aged 40 Years or Older, by Age Group and Sex for Those With Versus Without Schizophrenia in Ontario (With 6-Month Washout Period Applied to Annual Hip Fracture Counts)

Supplement 2.
Data Sharing Statement
