# Supplementary material for: Characterization of Hip Fractures Among Adults With Schizophrenia in Ontario, Canada
Source: JAMA Netw Open. 2023 Apr 28;6(4):e2310550. doi: 10.1001/jamanetworkopen.2023.10550 (PMC10148203; doi:10.1001/jamanetworkopen.2023.10550)
Supplement: Supplement 1. — eTable 1. Diagnosis and Physician Billing Codes eReferences eTable 2. Sex-Stratified Comparison of Sociodemographic and Clinical Characteristics of Individuals Aged 40 Years or Older, With Versus Without Schizophrenia, Who Sustained an Index Hip Fracture During Fiscal Years 2009 to 2018 in Ontario (N = 109 908) eTable 3. Sensitivity Analyses: Distribution of Hip Fracture Events and Crude Hip Fracture Rate of Individuals Aged 40 Years or Older, by Age Group and Sex for Those With Versus Without Schizophrenia in Ontario (With 6-Month Washout Period Applied to Annual Hip Fracture Counts) [file jamanetwopen-e2310550-s001.pdf]

Supplementary Online Content

Ansari H, Jaglal S, Cheung AM, Kurdyak P. Characterization of hip fractures among adults with schizophrenia in Ontario, Canada. *JAMA Netw Open*. 2023;6(4):e2310550. doi:10.1001/jamanetworkopen.2023.10550

eTable 1. Diagnosis and Physician Billing Codes

eReferences

eTable 2. Sex-Stratified Comparison of Sociodemographic and Clinical Characteristics of Individuals Aged 40 Years or Older, With Versus Without Schizophrenia, Who Sustained an Index Hip Fracture During Fiscal Years 2009 to 2018 in Ontario (N = 109 908)

eTable 3. Sensitivity Analyses: Distribution of Hip Fracture Events and Crude Hip Fracture Rate of Individuals Aged 40 Years or Older, by Age Group and Sex for Those With Versus Without Schizophrenia in Ontario (With 6-Month Washout Period Applied to Annual Hip Fracture Counts)

This supplementary material has been provided by the authors to give readers additional information about their work.

© 2023 Ansari H et al. *JAMA Network Open*.

eTable 1. Diagnosis and Physician Billing Codes

	**Diagnosis Codes**	**Billing codes**	**Data Source**
Fragility fractures [1,2]			
Hip fracture (1H in 1yr)	S72.0: head/neck of femur	n/a	DAD
	S72.1: pertrochanteric fracture		
	S72.2: subtrochanteric fracture		
Non-hip fragility fractures			
Spine (vertebra) (1H or 1P in 1yr)	S22.0: thoracic vertebra	805	DAD, NACRS, OHIP
	S22.1: thoracic spine (multiple fractures)		
	S32.0: lumbar vertebra		
	S32.7: lumbar spine and pelvis (multiple fractures)		
	S32.8: lumbar spine and pelvis (other/unspecified)		
Shoulder, upper arm, forearm (1H or 2P within 3mth in 1yr)	S42.2: upper humerus	812, 813	DAD, NACRS, OHIP
	S52.0-S52.6: forearm		
	S52.7: forearm (multiple)		
	S52.8: forearm (other parts)		
	S52.9: forearm (part unspecified)		
Pelvis (1H or 2P within 3mth in 1yr)	S32.1: sacrum	808	DAD, NACRS, OHIP
	S32.3: ilium		
	S32.4: acetabulum		
	S32.5: pubis		
	S32.7: lumbar spine and pelvis (multiple)		
	S32.8: lumbar spine and pelvis (other/unspecified)		
Schizophrenia [3]	F20, F25, F29	295, 298	DAD, OHIP, OMHRS
Hip Fracture Surgery [4]	n/a	F100: Fixation	DAD, OHIP
		F096: Intramedullary nail	
		F101, R440 or R439: Arthroplasty	
		CCI codes: IVA53, 1VA74, 1VA80, 1VC74, 1VC80	

Abbreviations (H: hospitalization; P: outpatient visit; CCI: Canadian Classification of Health Interventions)

© 2023 Ansari H et al. *JAMA Network Open*.

eReferences

1. Papaioannou A, Kennedy CC, Ioannidis G, et al. Comparative trends in incident fracture rates for all long-term care and community-dwelling seniors in Ontario, Canada, 2002–2012. *Osteoporosis International.* 2016;27(3):887-897.
2. O'Donnell S. Use of administrative data for national surveillance of osteoporosis and related fractures in Canada: Results from a feasibility study. *Archives of Osteoporosis.* 2013;8(1-2).
3. Kurdyak P, Lin E, Green D, Vigod S. Validation of a Population-Based Algorithm to Detect Chronic Psychotic Illness. *Canadian journal of psychiatry Revue canadienne de psychiatrie.* 2015;60(8):362-368.
4. Pincus D, Ravi B, Wasserstein D, et al. Association between wait time and 30-day mortality in adults undergoing hip fracture surgery. *JAMA.* 2017;318(20):1994-2003.

© 2023 Ansari H et al. *JAMA Network Open*.

eTable 2. Sex-Stratified Comparison of Sociodemographic and Clinical Characteristics of Individuals Aged 40 Years or Older, With Versus Without Schizophrenia, Who Sustained an Index Hip Fracture During Fiscal Years 2009 to 2018 in Ontario (N = 109 908)

	Males				Females			
**Characteristic**	**All males**	**No SCZ**	**Yes SCZ**	**Std Diff**	**All females**	**No SCZ**	**Yes SCZ**	**Std Diff**
	**N=(34,500)**	**N=(33,012)**	**N=(1,488)**		**N=(75,408)**	**N=(72,645)**	**N=(2,763)**	
	No. (%)	No. (%)	No. (%)		No. (%)	No. (%)	No. (%)	
**SOCIO-DEMOGRAPHIC**								
Age								
Mean(SD)	77.64 (12.5)	77.88 (12.4)	72.35 (12.9)	0.436	81.86 (10.4)	81.98 (10.3)	78.50 (11.5)	0.319
Median(Q1-Q3)	81 (70-87)	81 (71-87)	73 (62-83)	0.456	84 (77-89)	84 (77-89)	80 (71-87)	0.316
Rural/Small Town								
Missing	160 (0.5%)	150 (0.5%)	10 (0.7%)	0.029	307 (0.4%)	298 (0.4%)	9 (0.3%)	0.014
No	29,571 (85.7%)	28,233 (85.5%)	1,338 (89.9%)	0.134	65,038 (86.2%)	62,556 (86.1%)	2,482 (89.8%)	0.114
Yes	4,769 (13.8%)	4,629 (14.0%)	140 (9.4%)	0.144	10,063 (13.3%)	9,791 (13.5%)	272 (9.8%)	0.113
LTC-dwelling (pre-fracture)	4,790 (13.9%)	4,350 (13.2%)	440 (29.6%)	0.408	12,577 (16.7%)	11,573 (15.9%)	1,004 (36.3%)	0.478
Neighborhood income quintile								
Missing	252 (0.7%)	234 (0.7%)	18 (1.2%)	0.051	512 (0.7%)	498 (0.7%)	14 (0.5%)	0.023
1 (lowest)	8,256 (23.9%)	7,738 (23.4%)	518 (34.8%)	0.252	18,128 (24.0%)	17,293 (23.8%)	835 (30.2%)	0.145
2	7,131 (20.7%)	6,831 (20.7%)	300 (20.2%)	0.013	16,087 (21.3%)	15,508 (21.3%)	579 (21.0%)	0.01
3	6,592 (19.1%)	6,338 (19.2%)	254 (17.1%)	0.055	14,204 (18.8%)	13,741 (18.9%)	463 (16.8%)	0.056
4	6,269 (18.2%)	6,070 (18.4%)	199 (13.4%)	0.137	13,546 (18.0%)	13,084 (18.0%)	462 (16.7%)	0.034
5 (highest)	6,000 (17.4%)	5,801 (17.6%)	199 (13.4%)	0.116	12,931 (17.1%)	12,521 (17.2%)	410 (14.8%)	0.065
Instability Quintile								
Missing	481 (1.4%)	448 (1.4%)	33 (2.2%)	0.065	917 (1.2%)	868 (1.2%)	49 (1.8%)	0.048
1 (lowest)	3,753 (10.9%)	3,649 (11.1%)	104 (7.0%)	0.142	7,374 (9.8%)	7,165 (9.9%)	209 (7.6%)	0.082
2	5,188 (15.0%)	5,020 (15.2%)	168 (11.3%)	0.116	10,500 (13.9%)	10,199 (14.0%)	301 (10.9%)	0.095
3	6,298 (18.3%)	6,101 (18.5%)	197 (13.2%)	0.144	13,127 (17.4%)	12,727 (17.5%)	400 (14.5%)	0.083
4	7,674 (22.2%)	7,397 (22.4%)	277 (18.6%)	0.094	17,047 (22.6%)	16,414 (22.6%)	633 (22.9%)	0.008
5 (highest)	11,106 (32.2%)	10,397 (31.5%)	709 (47.6%)	0.335	26,443 (35.1%)	25,272 (34.8%)	1,171 (42.4%)	0.156

© 2023 Ansari H et al. *JAMA Network Open*.

Dependency Quintile								
Missing	481 (1.4%)	448 (1.4%)	33 (2.2%)	0.065	917 (1.2%)	868 (1.2%)	49 (1.8%)	0.048
1 (lowest)	3,835 (11.1%)	3,638 (11.0%)	197 (13.2%)	0.068	7,401 (9.8%)	7,107 (9.8%)	294 (10.6%)	0.028
2	4,787 (13.9%)	4,560 (13.8%)	227 (15.3%)	0.041	9,720 (12.9%)	9,325 (12.8%)	395 (14.3%)	0.043
3	5,709 (16.5%)	5,468 (16.6%)	241 (16.2%)	0.01	11,396 (15.1%)	10,991 (15.1%)	405 (14.7%)	0.013
4	6,743 (19.5%)	6,482 (19.6%)	261 (17.5%)	0.054	14,352 (19.0%)	13,841 (19.1%)	511 (18.5%)	0.014
5 (highest)	12,945 (37.5%)	12,416 (37.6%)	529 (35.6%)	0.043	31,622 (41.9%)	30,513 (42.0%)	1,109 (40.1%)	0.038
Deprivation Quintile								
Missing	481 (1.4%)	448 (1.4%)	33 (2.2%)	0.065	917 (1.2%)	868 (1.2%)	49 (1.8%)	0.048
1 (lowest)	5,885 (17.1%)	5,698 (17.3%)	187 (12.6%)	0.132	12,643 (16.8%)	12,253 (16.9%)	390 (14.1%)	0.076
2	6,432 (18.6%)	6,201 (18.8%)	231 (15.5%)	0.087	14,135 (18.7%)	13,700 (18.9%)	435 (15.7%)	0.082
3	6,625 (19.2%)	6,373 (19.3%)	252 (16.9%)	0.062	14,454 (19.2%)	13,930 (19.2%)	524 (19.0%)	0.005
4	6,973 (20.2%)	6,681 (20.2%)	292 (19.6%)	0.015	15,638 (20.7%)	15,070 (20.7%)	568 (20.6%)	0.005
5 (highest)	8,104 (23.5%)	7,611 (23.1%)	493 (33.1%)	0.226	17,621 (23.4%)	16,824 (23.2%)	797 (28.8%)	0.13
Ethnic Concentration Quintile								
Missing	481 (1.4%)	448 (1.4%)	33 (2.2%)	0.065	917 (1.2%)	868 (1.2%)	49 (1.8%)	0.048
1 (lowest)	7,665 (22.2%)	7,421 (22.5%)	244 (16.4%)	0.154	16,482 (21.9%)	16,013 (22.0%)	469 (17.0%)	0.128
2	7,534 (21.8%)	7,254 (22.0%)	280 (18.8%)	0.078	16,812 (22.3%)	16,267 (22.4%)	545 (19.7%)	0.065
3	6,614 (19.2%)	6,325 (19.2%)	289 (19.4%)	0.007	14,881 (19.7%)	14,324 (19.7%)	557 (20.2%)	0.011
4	6,380 (18.5%)	6,069 (18.4%)	311 (20.9%)	0.063	13,943 (18.5%)	13,340 (18.4%)	603 (21.8%)	0.086
5 (highest)	5,826 (16.9%)	5,495 (16.6%)	331 (22.2%)	0.142	12,373 (16.4%)	11,833 (16.3%)	540 (19.5%)	0.085
**CLINICAL / HEALTHCARE**								
Received hip fracture surgery	31,808 (92.2%)	30,450 (92.2%)	1,358 (91.3%)	0.035	70,646 (93.7%)	68,103 (93.7%)	2,543 (92.0%)	0.067
Index HF episode was coded as MRDx	32,369 (93.8%)	30,988 (93.9%)	1,381 (92.8%)	0.043	72,361 (96.0%)	69,759 (96.0%)	2,602 (94.2%)	0.086
Type of index hip fracture								
Head/neck of femur	16,839 (48.8%)	16,127 (48.9%)	712 (47.8%)	0.02	36,311 (48.2%)	35,024 (48.2%)	1,287 (46.6%)	0.033
Pertrochanteric	14,248 (41.3%)	13,638 (41.3%)	610 (41.0%)	0.006	32,720 (43.4%)	31,506 (43.4%)	1,214 (43.9%)	0.011
Subtrochanteric	1,629 (4.7%)	1,558 (4.7%)	71 (4.8%)	0.002	3,992 (5.3%)	3,874 (5.3%)	118 (4.3%)	0.05

© 2023 Ansari H et al. *JAMA Network Open*.

Other concurrent fracture with index HF episode								
Vertebral fracture	274 (0.8%)	260 (0.8%)	14 (0.9%)	0.017	258 (0.3%)	245 (0.3%)	13 (0.5%)	0.021
Pelvis fracture	369 (1.1%)	348 (1.1%)	21 (1.4%)	0.032	647 (0.9%)	618 (0.9%)	29 (1.0%)	0.021
Upper limb fracture	853 (2.5%)	809 (2.5%)	44 (3.0%)	0.031	3,050 (4.0%)	2,952 (4.1%)	98 (3.5%)	0.027
History of hip fracture	324 (0.9%)	309 (0.9%)	15 (1.0%)	0.007	1,091 (1.4%)	1,041 (1.4%)	50 (1.8%)	0.03
History of non-hip fragility fracture	4,213 (12.2%)	3,955 (12.0%)	258 (17.3%)	0.152	17,008 (22.6%)	16,268 (22.4%)	740 (26.8%)	0.102
History of either hip or non-hip fragility fracture	4,454 (12.9%)	4,187 (12.7%)	267 (17.9%)	0.146	17,694 (23.5%)	16,920 (23.3%)	774 (28.0%)	0.108
Frailty (from John Hopkins System)	11,535 (33.4%)	10,774 (32.6%)	761 (51.1%)	0.382	26,894 (35.7%)	25,374 (34.9%)	1,520 (55.0%)	0.412
No. of medical ADGs								
Mean (SD)	8.61 (3.84)	8.61 (3.82)	8.62 (4.18)	0.002	8.26 (3.68)	8.25 (3.67)	8.51 (4.01)	0.069
Median (Q1-Q3)	9 (6-11)	9 (6-11)	9 (5-12)	0.001	8 (5-11)	8 (5-11)	8 (5-12)	0.056
No. of psychosocial ADGs								
0	16,757 (48.6%)	16,548 (50.1%)	209 (14.0%)	0.838	34,679 (46.0%)	34,300 (47.2%)	379 (13.7%)	0.781
1	11,406 (33.1%)	10,867 (32.9%)	539 (36.2%)	0.07	27,340 (36.3%)	26,296 (36.2%)	1,044 (37.8%)	0.033
2	5,461 (15.8%)	4,883 (14.8%)	578 (38.8%)	0.564	11,845 (15.7%)	10,740 (14.8%)	1,105 (40.0%)	0.589
3	876 (2.5%)	714 (2.2%)	162 (10.9%)	0.359	1,544 (2.0%)	1,309 (1.8%)	235 (8.5%)	0.307
Rostered to a primary care provider								
0	1,767 (5.1%)	1,627 (4.9%)	140 (9.4%)	0.174	3,013 (4.0%)	2,810 (3.9%)	203 (7.3%)	0.152
1	27,026 (78.3%)	26,002 (78.8%)	1,024 (68.8%)	0.228	60,776 (80.6%)	58,785 (80.9%)	1,991 (72.1%)	0.21
2	5,707 (16.5%)	5,383 (16.3%)	324 (21.8%)	0.14	11,619 (15.4%)	11,050 (15.2%)	569 (20.6%)	0.141

Abbreviations (Std Diff: standardized difference; SD: standard deviation; Q1-Q3: quartile 1 – quartile 3; LTC: long-term care; MRDx: most responsible diagnosis; ADGs: Aggregated Diagnosis Groups)

© 2023 Ansari H et al. *JAMA Network Open*.

eTable 3. Sensitivity Analyses: Distribution of Hip Fracture Events and Crude Hip Fracture Rate of Individuals Aged 40 Years or Older, by Age Group and Sex for Those With Versus Without Schizophrenia in Ontario (With 6-Month Washout Period Applied to Annual Hip Fracture Counts)

	**Individuals with schizophrenia**			**Individuals without schizophrenia**		
**Age, y**	**Observed hip fracture events**	**Population (40+)**	**Crude rate per 10,000 (95% CI)**	**Observed hip fracture events**	**Population (40+)**	**Crude rate per 10,000 (95% CI)**
**Males**						
40-54	158	266551	5.9 (5.0-6.9)	2009	14858749	1.3 (1.3-1.4)
55-64	299	143853	20.8 (18.5-23.3)	3445	8564360	4.0 (3.8-4.1)
65-79	571	93738	60.9 (56.0-66.1)	10283	7175673	14.1 (13.9-14.4)
80+	542	34467	157.3 (144.3-171.1)	19103	2136854	87.9 (86.7-89.2)
All	**1,570**	**538609**	**29.2 (27.7-30.6)**	**38840**	**32735636**	**10.5 (10.4-10.6)**
**Females**						
40-54	81	223688	3.6 (2.9-4.5)	1360	15101811	0.9 (0.8-0.9)
55-64	295	155886	18.9 (16.8-21.2)	4305	8943473	4.8 (4.6-4.9)
65-79	1,036	133291	77.7 (73.1-82.6)	19123	8108380	23.3 (22.9-23.6)
80+	1,564	76530	204.4 (194.4-214.8)	53133	3436902	151.6 (150.3-152.9)
All	**2,976**	**589395**	**50.5 (48.7-52.3)**	**77291**	**35590566**	**21.5 (21.4-21.7)**
**Both sexes**						
40-54	239	490239	4.9 (4.3-5.5)	3369	29960560	1.1 (1.1-1.2)
55-64	594	299739	19.8 (18.3-21.5)	7750	17507833	4.4 (4.3-4.5)
65-79	1,607	227029	70.8 (67.4-74.3)	29406	15284053	19.0 (18.8-19.2)
80+	2,106	110997	189.7 (181.7-198.0)	72236	5573756	127.2 (126.3-128.1)
All	**4,546**	**1128004**	**40.3 (39.1-41.5)**	**112761**	**68326202**	**16.2 (16.1-16.3)**

Abbreviations (CI: confidence interval)

© 2023 Ansari H et al. *JAMA Network Open*.
